# Residential greenness is associated with disease severity among COVID-19 patients aged over 45 years in Wuhan, China

**DOI:** 10.1016/j.ecoenv.2022.113245

**Published:** 2022-03-01

**Authors:** Wenjia Peng, Haidong Kan, Lian Zhou, Weibing Wang

**Affiliations:** a School of Public Health, Shanghai Institute of Infectious Disease and Biosecurity, Fudan University, Shanghai, China; b Key Laboratory of Public Health Safety (Ministry of Education), Fudan University, Shanghai, China; c Jiangsu Provincial Center for Disease Control and Prevention, Nanjing, China; d IRDR-ICoE on Risk Interconnectivity and Governance on Weather/Climate Extremes Impact and Public Health, Fudan University, Shanghai, China

**Keywords:** COVID-19, Greenness, Air pollutant, Disease severity

## Abstract

Evidence regarding environmental factors associated with disease severity of COVID-19 remained scarce. This study aimed to investigate the association of residential greenness exposure with COVID-19 severity applying a retrospective cross-sectional study in Wuhan, China. We included 30,253 COVID-19 cases aged over 45 years from January 1 to February 27, 2020. Residential greenness was quantitatively assessed using normalized difference vegetation index (NDVI) and enhanced vegetation index (EVI). A multilevel generalized linear model using Poisson regression was implemented to analyze the association between greenness exposure and disease severity of COVID-19, after adjusting for potential covariates. A linear exposure-response relationship was found between greenness and COVID-19 severity. In the adjusted model, one 0.1 unit increase of NDVI and EVI in the 1000-m buffer radius was significantly associated with a 7.6% (95% confidence interval (CI): 4.0%, 11.1%) and 10.0% (95% CI: 5.1%, 14.7%) reduction of the prevalence of COVID-19 severity, respectively. The effect of residential greenness seemed to be more pronounced among participants with lower population density and economic levels. Air pollutants mediated 0.82~12.08% of the greenness and COVID-19 severity association, particularly to nitrogen dioxide. Sensitivity analyses suggested the robustness of the results. Our findings suggested that residential greenness exposure was beneficial to reduce the prevalence of COVID-19 severity.

## Highlights

• The study is the first to discuss the associations of greenness with COVID-19 severity.
• Greenness was associated with reduction of the prevalence of COVID-19 severity.
• The exposure-response relation between greenness and COVID-19 severity was linear.
• Air pollutants mediated the associations of greenness with COVID-19 severity.

## Graphical Abstract

**Figure:**
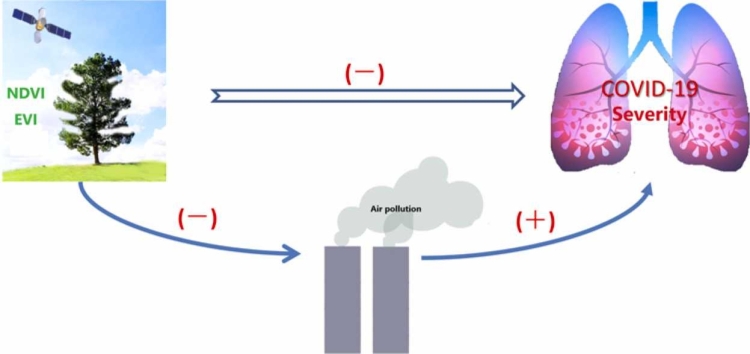

## 1 Introduction

Coronavirus disease 2019 (COVID-19), caused by a novel coronavirus (SARS-CoV-2), was first outbreak in Wuhan, China in December, 2019. On March 11, 2020, COVID-19 has officially been declared as an epidemic public health emergency of international concern by the World Health Organization (WHO) (Mahase, 2020), which has led to a total of 229,329,042 confirmed cases and 4,705,890 deaths detected in 223 countries as of September 20, 2021. The pandemic of COVID-19 has caused huge impacts on various aspects of society, including politics, economy, and culture. The epidemic is still raging in the European and American countries. According to Report of the WHO-China joint mission on COVID-19 (http://www.nhc.gov.cn), the clinical classification of COVID-19 is mainly divided into four types, namely mild, ordinary, severe, and critical based on the symptoms and imaging. The case fatality rate of severe and critical cases was much higher than non-severe ones (Guan et al., 2020). Therefore, investigating potential risk factors of COVID-19 severity is of great importance to prevent disease progression and adverse outcomes. Previous evidence had suggested several risk factors associated with COVID-19 disease severity, such as age, gender, diet and lifestyle habits, and underlying diseases, most of which are unalterable. Identifying modifiable factors for preventing COVID-19 progression are particularly important. Built environment is defined as the artificially constructed structures and infrastructure to provide for human activities, including land use, transport network, and greenness. The benefit effect of greenness on health outcome have been aroused great attention in recent years. A growing body of epidemiology studies has discussed the relationship between residential greenness and chronic non-infectious diseases, such as diabetes (Li et al., 2021, Yang et al., 2019b), hypertension (Jiang et al., 2021, Yang et al., 2019a) and cardiovascular diseases (Jia et al., 2018, Liu et al., 2021). Evidence regarding infectious diseases is still limited. Recently, two published ecological studies (Klompmaker et al., 2021, Russette et al., 2021) had suggested that exposure to greenness was associated with reduced county-level incidence and mortality in the United States, in which ecological fallacy was inevitable. Studies exploring the effect of greenness on infectious diseases, including COVID -19, are scarce at the individuals’ level.

Although the underlying mechanisms of greenness-associated health outcomes have not been fully elucidated, numerous studies have suggested several promising candidate pathways several pathways have suggested the rationalization of this association. First, green space can absorb air pollutants and purify the air (Hankey and Marshall, 2017). Second, contact with more greenness, such as public gardens, provided more opportunities to participate in physical activity (Sadeh et al., 2019). Third, exposure to more greenness is beneficial to relieve mental stress (Markevych et al., 2017).

To our knowledge, there are no studies that examined the association between residential greenness and disease severity among COVID-19 cases. To address the research gap, we employed a retrospective cross-sectional study to investigate whether a) higher residential greenness exposure was associated with reduced prevalence of COVID-19 severity; b) this association was modified by potential covariates; c) this association was mediated by air pollutants.

## 2 Methods

### 2.1 Setting and study population

Wuhan, the capital of Hubei province, is the central city in central China with the characteristic of south subtropic monsoon. It covers 8569.15 square kilometers of land area, with a permanent population of 12.3265 million in the seventh national census. Wuhan city consists of 13 districts, with a population ranging from 0.1355 to 1.6375 million. We retrospectively collected epidemiological data of COVID-19 cases which were confirmed by real-time reverse-transcriptase polymerase-chain-reaction (RT-PCR) and clinical-based diagnosis from January 1 to February 27, 2020 in Wuhan, China. A total of 47,103 COVID-19 cases were identified. We excluded the patients who were younger than 45 (n = 15,062). Among the remaining 32,041 patients, we excluded the subjects who reported travel history of Wuhan (n = 14), unspecific residential address (n = 1774). Finally, 30,253 participants aged 45 years and older from 169 streets were included in the final analysis. Only 3082 cases provided the data of preexisting comorbidities, of whom 1326 (43.02%) reported having at least one comorbidity. The most common comorbidities included hypertension (n = 773), diabetes (n = 326), cardiovascular diseases (n = 319) and lung disease (n = 111).

The Ethics Committee of the School of Public Health of Fudan University, Shanghai, approved this study. Written informed consent was obtained before the investigation.

### 2.2 Outcomes

All the COVID-19 patients were divided into mild, moderate, severe, and critical according to the report of the WHO-China Joint Mission on COVID-19 (China, 2020). We categorized the outcome into two groups: non-severity (mild and moderate) and severity group (severe and critical).

### 2.3 Greenness exposure

Greenness exposure was quantitatively assessed using normalized difference vegetation index (NDVI) and enhanced vegetation index (EVI), which were derived from the Terra Moderate Resolution Imaging Spectroradiometer (MODIS) Vegetation Indices products, namely MOD13Q1. MOD13Q1 data are generated every 16 days at 250-meter (m) spatial resolution. The algorithm chooses the best available pixel value from all the acquisitions, with low clouds, low view angle, and the highest NDVI or EVI value. The valid range of NDVI and EVI values is from − 0.2 to 1, with no unit. The closer that the values are to 1, the more greenness there is. A negative value indicates that the ground cover is cloud, water, or snow, which was set to 0. We downloaded three MOD13Q1product images with HDF format in June, July, and August 2019 (summer) at https://ladsweb.modaps.eosdis.nasa.gov/search/, as the greenest months in China. We also downloaded the images in the period of March 2019–May 2019, September 2019–November 2019, and December 2019–February 2020, representing the exposure level of spring, autumn, and winter, respectively. NDVI and EVI in the area of interest were extracted from the vegetation layers using the process of a crop by mask and scale factor. Individuals’ residential address was transformed into longitude and latitude coordinates. We calculated the average NDVI and EVI with 500-m, 1000-m, and 1500-m buffer radii around the corresponding residential address as the greenness based on zonal statistics and extract values to points. The calculation of greenness was completed using ArcGIS 10.4.

### 2.4 Potential covariates

According to prior knowledge, the following potential covariates were considered in our analysis: age, gender, days from symptom onset to diagnosis, street-level population density, and nighttime light. Age was categorized into two groups: 45–64 years and ≥ 65 years. The variable of days from symptom onset to diagnosis was dichotomized as < 10 and ≥ 10 according to the median. We downloaded a population density dataset in 2019 from the website of WorldPop available at https://www.worldpop.org/. The datasets are stored in Geotiff format at a resolution of approximately one kilometer (km). Nighttime light data was derived from the Visible Infrared Imaging Radiometer Suite (VIIRS) monthly composites as a proxy of economic level. We downloaded the image in the time of December 2019 from Earth Observation Group (https://eogdata.mines.edu/). Street-level population density and nighttime light were obtained using ArcGIS 10.4.

### 2.5 Candidate mediators

We considered air pollutants as candidate mediators according to the previous studies. Air pollutants including fine particulate matter (PM_2.5_), inhale particulate matter (PM_10_) and nitrogen dioxide (NO_2_) were collected from 21 monitoring stations of the Department of Ecological and Environment of Wuhan city (http://hbj.wuhan.gov.cn/). To obtain the individuals’ exposure level, we applied an inverse distance weighting (IDW) interpolation algorithm, as one of the most used interpolation methods described everywhere (Guo et al., 2020). Two main parameters, namely inverse distance weighting power and the number of points used for interpolation, were taken as 2 and 12 based on the default value of ArcGIS 10.4, respectively.

### 2.6 Statistical analysis

Quantitative data with normal distribution were expressed as mean and standard deviation, and differences between groups were compared using a two-independent samples t-test. Qualitative data were reported using numbers and rates, and differences between groups were compared using the Chi-square test. A generalized additive mixed model with penalized cubic regression splines was used to discuss whether the association between greenness exposure and COVID-19 severity was linear. A multilevel generalized linear model using Poisson regression with a random intercept for street (*jiedao*) was employed to analyze the association between greenness exposure in 1000-m buffer radius and disease severity of COVID-19 because the outcome was common. A prevalence ratio (PR) with its 95% confidence interval (CI) was reported.

Subgroup analyses by gender (male and female), age (45–64 and ≥ 65 years), days from symptom onset to diagnosis (< 10 and ≥ 10), population density (< median: 14,620.3 and ≥ median), and nighttime light (< median: 35.8 and ≥ median) were performed to discuss the susceptible population. The effect modification was tested by adding interaction as a product term into the model, which was adjusted for age, gender, days from symptom onset to diagnosis, population density, nighttime light, except for the interaction covariates. Mediation analysis was conducted to investigate whether the association between greenness and disease severity of COVID-19 was mediated by air pollutants (PM_2.5_, PM_10,_ and NO_2_). We estimated the total effects, direct effects, and indirect effects (mediation effect) by adjusting potential confounders. The significance of the mediation effect was tested through 500 bootstrap resamples of the estimated indirect effect. We reported the average direct effect (ADE) and average causal mediation effect (ACME).

To ensure the robustness of our results, we also performed three sensitivity analyses. First, we used NDVI and EVI values in other seasons (Spring, Autumn, and Winter) as the exposure measurement. Second, we replicated the above analyses in the other two buffer radii (500-m and 1500-m). Third, we limited the cases reporting at least one preexisting comorbidity (n = 1326).

All the statistical analyses were conducted in R statistical software, Version 4.0.3 (University of Auckland, New Zealand) using gamm4 packages (for cubic splines analysis), lme4 package (for main model analysis), or mediation packages (for mediation analysis).

## 3 Results

### 3.1 Characteristics of participants

**Fig. 1:**
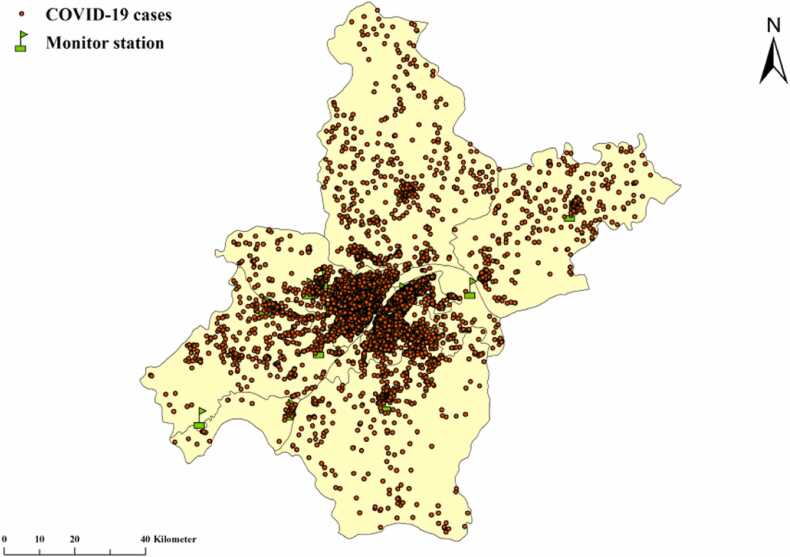
Spatial distribution of 30,253 COVID-19 cases and 21 environment monitoring stations in Wuhan.

Among the 30,253 eligible COVID-19 cases, 6173 severity cases (20.40%) were observed. The spatial distribution of all the cases was shown in Fig. 1. The basic characteristics, social-economic level as well as greenness indicators grouped by COVID-19 severity were presented in Table 1. In brief, COVID-19 severity was more likely to be male, older age, longer days from symptom onset to diagnosis, higher population density, and higher nighttime light. The average greenness indicators in summer were higher in non-severity cases compared to severity ones.

**Table 1:** Characteristics of participants by COVID-19 severity.

Variables	Total	Non-severity	Severity	$\chi^{2}/t$	*P*
Overall	30,253	24,080 (79.60)	6173 (20.40)	–	–
Gender				22.462	< 0.001
Male	14,233	11,163 (78.43)	3070 (21.57)		
Female	16,020	12,917 (80.63)	3103 (19.37)		
Age (years)				713.695	< 0.001
45–64	18,449	15,598 (84.55)	2851 (15.45)		
≥ 65	11,804	8482 (71.86)	3322 (28.14)		
Days				423.353	< 0.001
< 10	16,593	13,925 (83.92)	2668 (16.08)		
≥ 10	13,660	10,155 (74.34)	3505 (25.66)		
Population density(person/sq. km.)				14.519	< 0.001
< median (14,620.3)	15,357	12,357 (80.46)	3000 (19.54)		
≥ median (14,620.3)	14,896	11,723 (78.70)	3173 (21.30)		
Nighttime light				21.301	< 0.001
< median (35.8)	15,358	12,386 (80.65)	2972 (19.35)		
≥ median (35.8)	14,895	11,694 (78.51)	3201 (21.49)		
NDVI _500−m_	–	0.324 ± 0.117	0.309 ± 0.100	8.890	< 0.001
NDVI _1000−m_	–	0.319 ± 0.111	0.304 ± 0.095	9.921	< 0.001
NDVI _1500−m_	–	0.318 ± 0.109	0.301 ± 0.093	11.175	< 0.001
EVI _500−m_	–	0.191 ± 0.084	0.182 ± 0.073	7.749	< 0.001
EVI _1000−m_	–	0.187 ± 0.080	0.177 ± 0.068	9.043	< 0.001
EVI _1500−m_	–	0.185 ± 0.078	0.174 ± 0.067	10.176	< 0.001

### 3.2 Association between greenness and COVID-19 severity

**Fig. 2:**
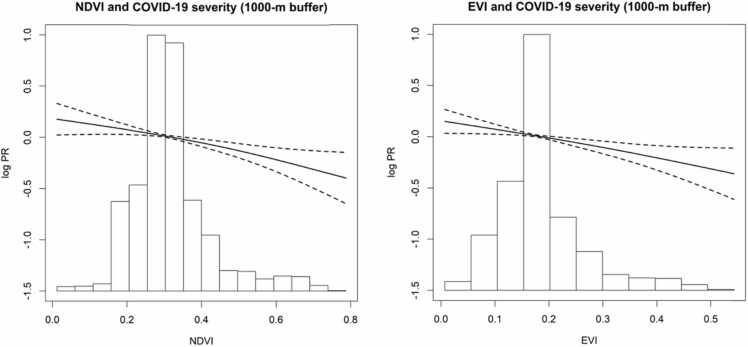
Exposure-response curves of the association between greenness in 1000-m buffer radius with COVID-19 severity. log PR: log prevalence ratio. The solid line shows the exposure-response curve, dotted lines show the 95% CI of the exposure-response curves, histograms of greenness distribution are shown on the x-axis. All the models were adjusted for age, gender, days from symptom onset to diagnosis, population density, nighttime light.

The overall exposure-response curves of the association between greenness in 1000-m buffer and COVID-19 severity showed almost linear (Fig. 2). In the adjusted models, one 0.1 unit increase in both NDVI and EVI within 1000-m buffer was associated with a 7.6% (PR: 0.924, 95% CI: 0.889, 0.960) and 10.0% (PR: 0.900, 95% CI: 0.853, 0.949) reduction of the prevalence of COVID-19 severity.

**Fig. 3:**
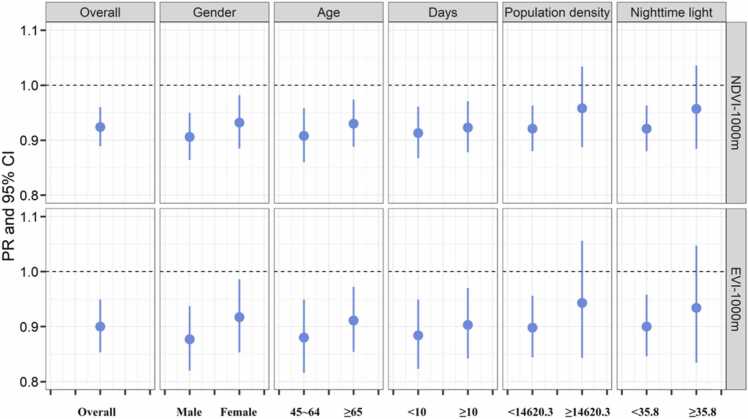
Stratified analyses on per 0.1 unit increase in NDVI and EVI in 1000-m buffer radius and COVID-19 severity. Except for the stratified covariates, all the stratified analyses were adjusted for age, gender, days from symptom onset to diagnosis, population density, nighttime light.

In the stratified analysis (Fig. 3), there were no obvious differences between gender, age, and days from symptom onset to diagnosis. When stratified by population density and nighttime light, we found the significant protective effect only in lower population density and lower nighttime light groups, although the interaction test did not reach the statistically significant level.

### 3.3 Mediation analysis by air pollutants

Table 2 showed that air pollutants mediated the association between greenness and COVID-19 severity. More specifically, PM_2.5_, PM_10,_ and NO_2_ significantly mediated 2.26%, 0.82%, and 12.08% of the total effects of NDVI on COVID-19 severity, respectively. Similarly, 1.18%, 0.89%, and 7.82% of the effects of EVI on COVID-19 severity were mediated by PM_2.5_, PM_10,_ and NO_2_, respectively.

**Table 2:** Mediation effect on the relationship between greenness and COVID-19 severity by air pollutants.^a^

Greenness	Mediator	ACME estimate (95% CI)	ADE estimate (95% CI)	Proportion mediated (95% CI) (%)	Proportion *P* value
NDVI_1000m_	PM_2.5_	-0.0004 (−0.0008, −0.0001)	-0.0182(−0.0308, −0.0075)	2.26 (0.64, 5.93)	< 0.01
	PM_10_	-0.0002 (−0.0004, −0.0000)	-0.0188(−0.0296, −0.0079)	0.82 (0.01,2.77)	0.05
	NO_2_	-0.0018 (−0.0026, −0.0009)	-0.0133 (−0.0241, −0.0030)	12.08 (4.50, 37.13)	< 0.01
EVI_1000m_	PM_2.5_	-0.0003 (−0.0005, −0.0001)	-0.0220 (−0.0387, −0.0087)	1.18 (0.20, 4.44)	0.01
	PM_10_	-0.0002 (−0.0005, −0.0000)	-0.0231 (−0.0376, −0.0092)	0.89 (0.04, 2.50)	0.04
	NO_2_	-0.0014 (−0.0023, −0.0007)	-0.0171 (−0.0323, −0.0032)	7.82 (2.66, 30.98)	0.01

a Adjusting age, gender, days from symptom onset to diagnosis, population density, nighttime light.

ACME, average causal mediation effect; ADE, average direct effect.

### 3.4 Sensitivity analysis

When we considered greenness in other buffer radii (500-m and 1500-m), the results from cubic spline, stratified and mediation effects analyses were similar to those in 1000-m buffer (Fig. S1-S4, Table S1-S2). The results were consistent when we used greenness in other seasons (spring, autumn, winter) as exposure measurement (Table S2). When we limited the cases reporting the preexisting comorbidities (n = 1326), we also detected a significant protective effect of NDVI (PR: 0.877, 95% CI: 0.784, 0.981) and EVI (PR: 0.850, 95% CI: 0.724, 0.998).

## 4 Discussion

### 4.1 Main findings and interpretation

There is no doubt that access to more green plays an important role in preserving human health. Recent studies suggested a beneficial effect of greenness on COVID-19 case rates. Specifically, Russette et al. (2021) and Klompmaker et al. (2021) both suggested that greenness by Leaf Area Index (LAI) and NDVI was negatively associated with COVID-19 mortality using an ecological study based on approximately 3000 counties in the United States. Similarly, Spotswood et al. (2021) suggested higher COVID-19 case rates in less-green neighborhoods in the United States. A study from 380 counties in Poland (Ciupa and Suligowski, 2021) also indicated that higher greenness explained lower COVID-19 deaths.

This retrospective cross-sectional study with larger sample size investigated the association between greenness exposure measured by vegetation indicators (NDVI and EVI) and disease severity of COVID-19 in Wuhan, as well as the potential mediation effect by air pollutants. We found that higher exposure to greenness was associated with reduced prevalence of COVID-19 severity. The exposure-response curve revealed this association was linear. Mediation effect analysis showed that this association was partially mediated by air pollutants, especially NO_2_. Consistent with the study (Klompmaker et al., 2021) linking the association between NDVI and COVID-19 mortality, we found that the exposure-response curve for disease severity of COVID-19 was inverse and linear.

Although stratified analyses by population density and nighttime light did not exhibit any statistical differences, we found that the protective effect of greenness on disease severity among patients with lower groups. The results were consistent with recent studies (McEachan et al., 2016, Sarkar et al., 2018) showing more pronounced in lower social-economic status. One possible reason is that individuals in deprived areas are generally less mobile, which promoted them contact with greenness frequently and social interaction with other neighborhoods (Maas et al., 2009).

The spatial scale is an important factor when investigating the association between greenness and health. A common approach is to construct a buffer around locations of interest. The best buffer size surrounding an individual’s home was inconclusive. The setting of buffer sizes was mainly based on the distance travel time. Browning and Lee (2017) reviewed 47 articles and suggested that buffer size within 1000–2000 m was recommended. Zhang et al. (2019b) also reported that the strongest association was observed at buffers smaller than 1600 m. Previous studies (Liu et al., 2021, Peng et al., 2021) on the association between greenness and health outcomes would use various buffer sizes to determine the robustness of the results in sensitivity analysis surrounding the residential address, and both of them derived similar results. We chose 500-m, 1000-m, and 1500-m buffer sizes around the residential address to measure the greenness exposure, corresponding to 5-minute, 10-min, and 15-min walking distances. We found the consistent protective effect of greenness in three buffer sizes. However, the magnitudes of the association in the 1500-m buffer (PR for NDVI and EVI: 0.915; 0.893) seemed to be stronger than 500-m (0.940; 0.926) and 1000-m (0.924; 0.900).

### 4.2 Potential mechanism

Recent studies (Jiang et al., 2021, Peng et al., 2021, Xie et al., 2021, Yang et al., 2021) conducting the association of greenness with health outcomes had attempted to uncover the potential mechanism through mediation analysis. One of the most widely discussed mediators was air pollutants, including particulate matters, NO_2_, etc. Similar to previous studies (Jiang et al., 2021, Yang et al., 2021), we also found that PM_2.5_, PM_10,_ and NO_2_ mediated the association between greenness and COVID-19 severity, particularly to NO_2_. Studies (Sepehri et al., 2020, Sepehri and Sarrafzadeh, 2019) suggested that this compound occurs in the aqueous media. The explanation of greenness—air pollutants—COVID-19 association seemed to be plausible. Exposure to more green is beneficial to reduce the level of ambient air pollutants. For example, Ozdemir (2019) suggested that planting roadside trees could significantly decrease the concentration of vehicle-related PM2.5 and heavy metal. Exposure to air pollutants was positively associated with incidence (Stieb et al., 2020), mortality (Wu et al., 2020), and case fatality rate (Tian et al., 2021) of COVID-19. Linares et al. (2021) indicated that NO_2_ exposure was more prominent to be associated with higher severity of COVID-19 compared with other pollutants. Increasing evidence has sought the potential mechanism of air pollutants and adverse outcomes of COVID-19. One explanation is that air pollutants could trigger oxidative stress and induce inflammation reactions, which may eventually deteriorate the immune system (Qin et al., 2020). On the other hand, long-term and short-term exposure to air pollutants were associated with hospitalization visits of many diseases (Chen et al., 2018, Tian et al., 2019, Yan et al., 2021), especially respiratory system diseases (Zhang et al., 2019). Comorbidities could accelerate the disease progression of COVID-19. In addition, Paital and Agrawal (2020) found that NO_2_ and PM_2.5_ exposure were associated with elevated expression of angiotensin-converting enzyme 2 (ACE-2), as a receptor for SARS-CoV2. Another important mediator, physical activity, has been discussed in many epidemiological studies. Living surrounding greener areas might promote individuals to participate in physical activity (Sadeh et al., 2019). Regular physical activity is beneficial for strengthening the immune system to protect against virus infection. In addition, greenness has been suggested to buffer the effects of traffic noise through acoustic (Markevych et al., 2017). Higher greenness may reduce noise level through either deflection or absorption of the noise. A recent ecological time-series study (Diaz et al., 2021) showed that noise pollution was related to the rate of intensive care unit admissions. However, due to the lack of physical activity and noise exposure, we are unable to analyze the mediation effect of these factors.

### 4.3 Strengths and limitations

There are several strengths in our study. First, this is the first study to quantitatively evaluate the association between greenness exposure and disease severity of COVID-19 with larger sample size. Second, a series of sensitivity analyses suggested the robustness of our results. Third, we estimated the indirect effect of air pollutants, which might be critical to in providing some shreds of evidence for revealing the potential mechanisms. Several limitations should be acknowledged in this study. First, this study is a retrospective cross-sectional study, making it impossible to derive a causation effect. Although we have adjusted a set of potential confounders based on prior knowledge, some unmeasured confounders, such as family income, education status, are not available in this study. However, we used a satellite-based nighttime light as a proxy of social-economic level. The nighttime light represents the artificial human lighting at night, directly showing human activities in a region through light distribution and brightness. Research have confirmed a close relationship between satellite-based nighttime light and GDP (Bennett and Smith, 2017). Second, we did not consider population mobility when measuring individuals’ exposure to environmental factors. In other words, we assumed that all COVID-19 patients were permanent residents, at least in the recent past. To minimize the mobility, we only included cases aged 45 years and old, namely middle-aged, and older adults. Cases reporting the travel history in Wuhan were excluded from our study. Third, NDVI/EVI-based greenness only reflects the total amount of vegetation in an area, and could not differentiate the composition of greenness (e.g. parks) and the type of vegetation (e.g. shrubs, grass, canopy). Several studies (Astell-Burt and Feng, 2019, Zhang and Tan, 2019a) suggested a stronger association of canopy cover with health outcomes. Fourth, there was a large number of missing values on preexisting comorbidities, reaching 88.81%. The prevalence of preexisting comorbidities might be bias to represent the overall population. Thus, we did not consider the preexisting comorbidities as a covariate in the main analysis. However, in sensitivity analysis, we found the protective effect of greenness remained significant when limited to the cases reporting at least one comorbidity. Fifth, to obtain the individuals’ exposure to air pollutants, we applied a spatial interpolation based on the IDW algorithm, which is mainly dependent on the spatial distribution of environmental monitoring stations. Most of the monitor stations were located in the central urban area, which also reported the majority of cases. Based on the above limitations, the results derived should be interpreted with caution. More well-designed studies are necessary to validate our results.

## 5 Conclusion

In summary, residential greenness was associated with decreased prevalence of COVID-19 severity, highlighting the impact of environmental factors on infectious diseases. Mediation analysis indicated that this association was partially mediated by air pollutants, especially NO_2_. These results might be important toward increasing more green for policymakers, curbing disease progression of COVID-19. The underlying mechanisms between greenness and disease severity of COVID-19 still require further researches.

## Funding

This study was granted by 10.13039/100000865Bill & Melinda Gates Foundation, Seattle, WA, United States (Grant No. INV-006277), 10.13039/501100001809National Natural Science Foundation of China, China (Grant No. 82073612), and Shanghai New Three-year Action Plan for Public Health, China (Grant No. GWV-10.1-XK16).

## CRediT authorship contribution statement

**Wenjia Peng:** Conceptualization, Software, Formal analysis, Writing – original draft. **Haidong Kan:** Conceptualization, Resources. **Lian Zhou:** Conceptualization, Resources. **Weibing Wang:** Conceptualization, Resources, Writing – review & editing, Project administration, Funding acquisition.

## Declaration of Competing Interest

The authors declare that they have no known competing financial interests or personal relationships that could have appeared to influence the work reported in this paper.
