# Supplementary material for: Residential greenness is associated with disease severity among COVID-19 patients aged over 45 years in Wuhan, China
Source: Ecotoxicol Environ Saf. 2022 Mar 1;232:113245. doi: 10.1016/j.ecoenv.2022.113245 (PMC8786605; doi:10.1016/j.ecoenv.2022.113245)
Supplement: Supplementary file 1 — Supplementary material [file mmc1.docx]

# Residential greenness is associated with disease severity among COVID-19 patients aged over 45 years in Wuhan, China

Wenjia Peng ^a,b^, Haidong Kan^a^, Lian Zhou^c^, Weibing Wang ^a,b,d^

^a^ School of Public Health, Shanghai Institute of Infectious Disease and Biosecurity, Fudan University, Shanghai, China.

^b^ Key Laboratory of Public Health Safety (Ministry of Education), Fudan University, Shanghai, China.

^c^ Jiangsu Provincial Center for Disease Control and Prevention, Nanjing, China

^d^ IRDR-ICoE on Risk Interconnectivity and Governance on Weather/Climate Extremes Impact and Public Health, Fudan University, Shanghai, China.

Corresponding author: Weibing Wang, Department of Epidemiology, School of Public Health, Fudan University (E-mail: [wwb@fudan.edu.cn](mailto:wwb@fudan.edu.cn))

# Supplementary tables and figures


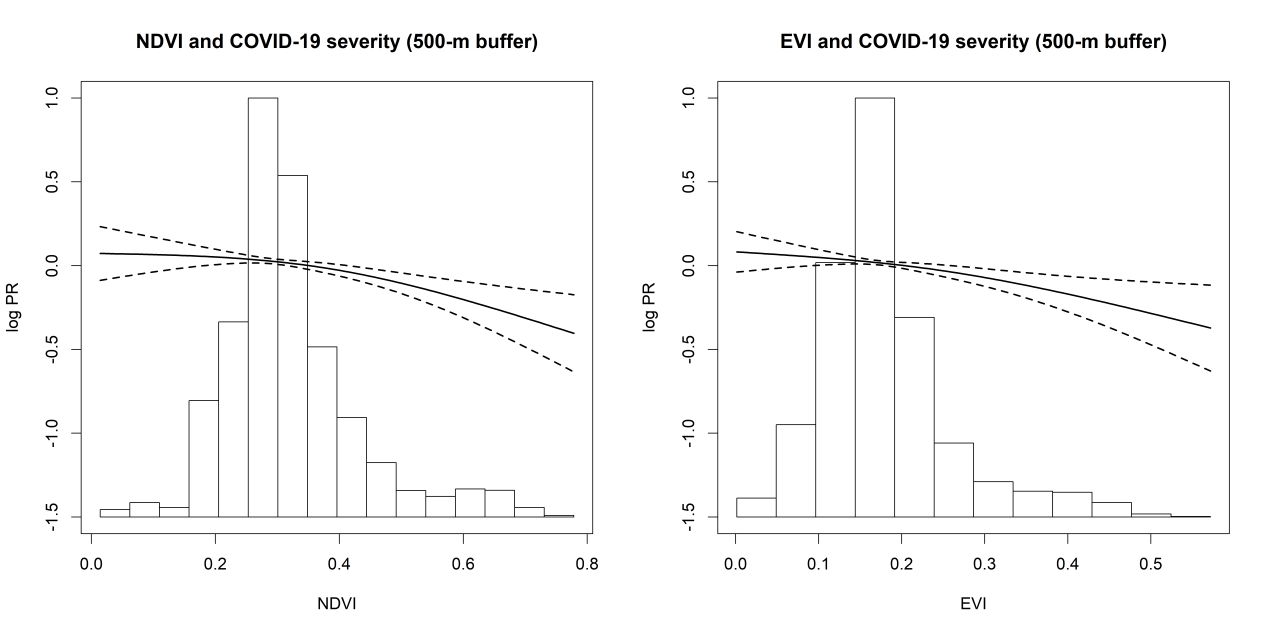


Figure S1 Exposure-response curves of the association between greenness in 500-m buffer radius with COVID-19 severity. log PR: log prevalence ratio. Solid line shows the exposure-response curve, dotted lines show the 95% CI of the exposure-response curves, histograms of greenness distribution are shown on x-axis. All the models were adjusted for age, gender, days from symptom onset to diagnosis, population density, nighttime light.


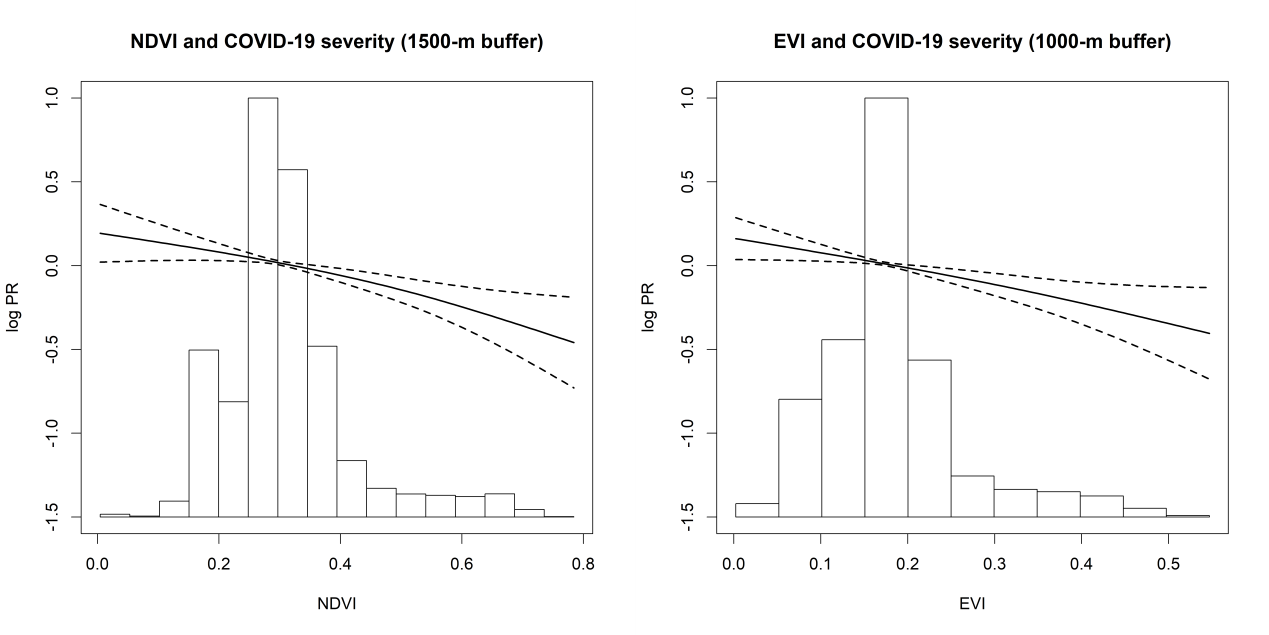


Figure S2 Exposure-response curves of the association between greenness in 1500-m buffer radius with COVID-19 severity. log PR: log prevalence ratio. Solid line shows the exposure-response curve, dotted lines show the 95% CI of the exposure-response curves, histograms of greenness distribution are shown on x-axis. All the models were adjusted for age, gender, days from symptom onset to diagnosis, population density, nighttime light.


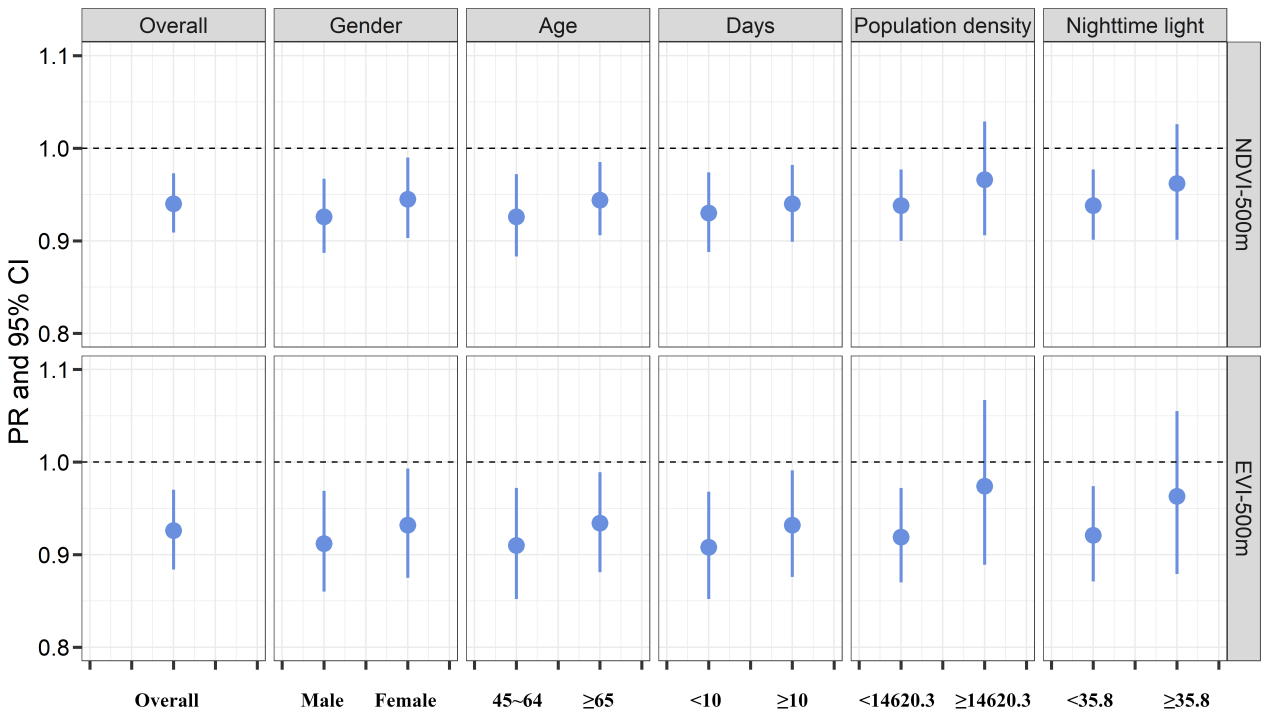


Figure S3 Stratified analyses on per 0.1 unit increase in NDVI and EVI in 500-m buffer radius and COVID-19 severity. Except for the stratified covariates, all the stratified analyses were adjusted for age, gender, days from symptom onset to diagnosis, population density, nighttime light.


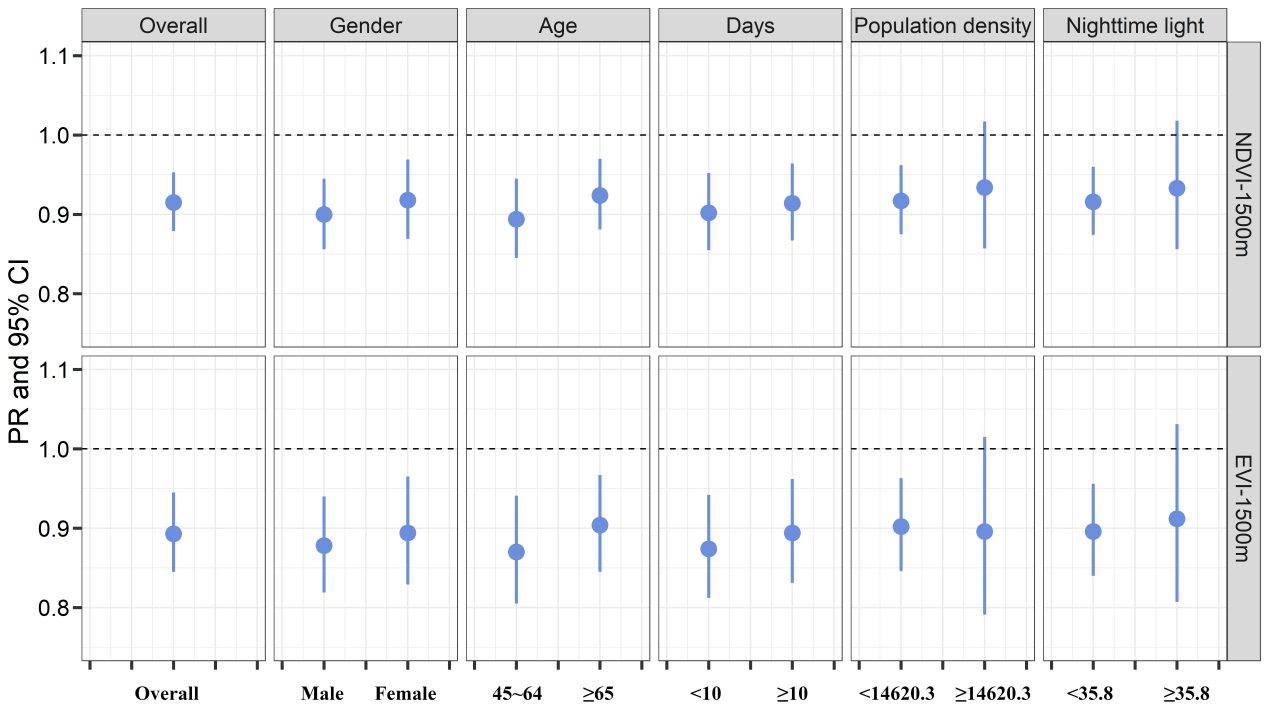


Figure S4 Stratified analyses on per 0.1 unit increase in NDVI and EVI in 500-m buffer radius and COVID-19 severity. Except for the stratified covariates, all the stratified analyses were adjusted for age, gender, days from symptom onset to diagnosis, population density, nighttime light.

Table S1 Mediation effect on the relationship between greenness and COVID-19 severity by air pollutants

| Greenness | Mediator | ACME estimate  (95% CI) | ADE estimate  (95% CI) | Proportion mediated  (95% CI) (%) | Proportion *P* value |
| --- | --- | --- | --- | --- | --- |
| NDVI_500m_ | PM_2.5_ | -0.0003 (-0.0005, -0.0001) | -0.0136 (-0.0232, -0.0054) | 2.09 (0.60, 5.80) | <0.001 |
|  | PM_10_ | -0.0001 (-0.0003, -0.0000) | -0.0144 (-0.0245, -0.0059) | 0.96 (0.64, 2.90) | 0.030 |
|  | NO_2_ | -0.0012 (-0.0018, -0.0006) | -0.0105(-0.0206, -0.0016) | 10.60 (4.01, 41.16) | <0.001 |
| EVI_500m_ | PM_2.5_ | -0.0001 (-0.0002, 0.0000) | -0.0160 (-0.0280, -0.0046) | 0.49 (-0.41, 2.38) | 0.20 |
|  | PM_10_ | -0.0002 (-0.0004, -0.0000) | -0.0167(-0.0293, -0.0054) | 1.21 (0.17, 3.99) | 0.03 |
|  | NO_2_ | -0.0009 (-0.0015, -0.0004) | -0.0122 (-0.0225, -0.0012) | 6.65 (2.28, 36.15) | 0.02 |
| NDVI_1500m_ | PM_2.5_ | -0.0004 (-0.0007, -0.0001) | -0.0209 (-0.0347, -0.0103) | 1.79 (0.44, 4.73) | 0.02 |
|  | PM_10_ | -0.0002 (-0.0004, 0.0003) | -0.0220 (-0.0354, -0.0106) | 0.73 (-0.17, 2.35) | 0.12 |
|  | NO_2_ | -0.0018 (-0.0028, -0.0009) | -0.0153 (-0.0295, -0.0043) | 10.54 (3.70, 31.29) | <0.001 |
| EVI_1500m_ | PM_2.5_ | -0.0001 (-0.0003, 0.0000) | -0.0239 (-0.0395, -0.0097) | 0.43 (-0.23, 1.87) | <0.001 |
|  | PM_10_ | -0.0001 (-0.0004, 0.0000) | -0.0251 (-0.0416, -0.0104) | 0.46 (-0.02, 1.81) | 0.09 |
|  | NO_2_ | -0.0014 (-0.0022, -0.0007) | -0.0176(-0.0333, -0.0033) | 7.55 (2.78, 33.21) | <0.001 |

^a^ Adjusting age, gender, days from symptom onset to diagnosis, population density, nighttime light，

ACME, average causal mediation effect; ADE, average direct effect.

Table S2 Sensitivity analysis for greenness in other seasons and buffer radius

| Analysis | NDVI | |  | EVI | |
| --- | --- | --- | --- | --- | --- |
| Season |  | |  |  | |
| Spring | 0.936 (0.900, 0.973) | <0.001 |  | 0.905 (0.850, 0.963) | 0.002 |
| Autumn | 0.952 (0.918, 0.988) | 0.010 |  | 0.917 (0.862, 0.975) | 0.006 |
| Winter | 0.940 (0.898, 0.985) | 0.009 |  | 0.866 (0.786, 0.953) | 0.003 |
| Buffer radius |  |  |  |  |  |
| 500-m | 0.940 (0.909, 0.972) | <0.001 |  | 0.925 (0.884, 0.969) | 0.001 |
| 1500-m | 0.915 (0.879, 0.953) | <0.001 |  | 0.893 (0.845, 0.945) | <0.001 |
